# 3-Meth­oxy-2-[2-({[6-(trifluoro­meth­yl)pyridin-2-yl]­oxy}meth­yl)phen­yl]prop-2-enoic acid

**DOI:** 10.1107/S1600536812042316

**Published:** 2012-10-20

**Authors:** Rajni Kant, Vivek K. Gupta, Kamini Kapoor, Chetan S. Shripanavar, Madhukar B. Deshmukh, Kaushik Banerjee

**Affiliations:** a X-ray Crystallography Laboratory, Post-Graduate Department of Physics & Electronics, University of Jammu, Jammu Tawi 180 006, India; b National Research Centre for Grapes, Pune 412307, India; c Department of Chemistry, Shivaji University, Kolhapur, 416 004, India

## Abstract

The title mol­ecule, C_17_H_14_F_3_NO_4_, consists of two nearly planar fragments, *viz.* the 2-benzyl­oxypyridine (r.m.s. deviation 0.016 Å) and (*E*)-3-meth­oxy­prop2-enoic (r.m.s. deviation 0.004 Å) units, which form a dihedral angle of 84.19 (7)°. In the crystal, pairs of O—H⋯O hydrogen bonds link mol­ecules into dimers that are further connected by C—H⋯O and C—H⋯F inter­actions into (001) layers. In addition, π–π stacking inter­actions are observed within a layer between the pyridine and benzene rings [centroid–centroid distance = 3.768 (2) Å]. The F atoms of the trifluoro­methyl group are disordered over two sets of sites in a 0.53 (4):0.47 (4) ratio.

## Related literature

The title compound is the acid metabolite of picoxystrobin [systematic name: methyl (*E*)-3-meth­oxy-2-{2-[6-(trifluoro­meth­yl)-2-pyridyl­oxymeth­yl]phen­yl}acrylate], a systemic fungicide with broad-spectrum bio-efficacy against various diseases of agricultural crops, see: Balba (2007 ▶); Ammermann *et al.* (2000 ▶). For a related structure, see: Kant *et al.* (2012 ▶).
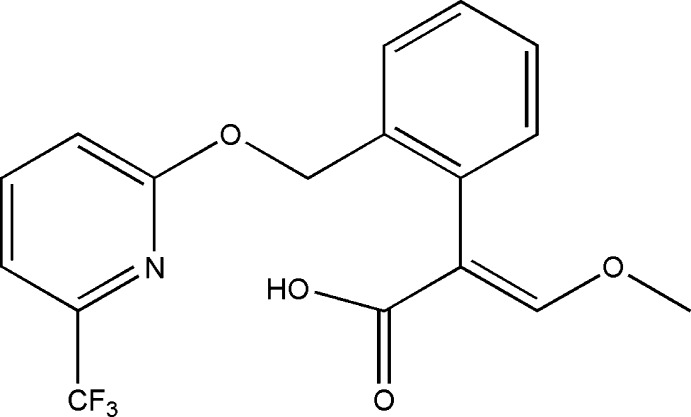


## Experimental

### 

#### Crystal data

C_17_H_14_F_3_NO_4_
*M* *_r_* = 353.29
Triclinic, 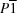

*a* = 7.4701 (4) Å
*b* = 10.1619 (5) Å
*c* = 11.8219 (5) Å
α = 94.721 (4)°
β = 100.079 (4)°
γ = 110.685 (5)°
*V* = 816.42 (7) Å^3^
*Z* = 2
Mo *K*α radiation
μ = 0.13 mm^−1^
*T* = 293 K
0.3 × 0.2 × 0.2 mm

#### Data collection

Oxford Diffraction Xcalibur Sapphire3 diffractometer
Absorption correction: multi-scan (*CrysAlis PRO*; Oxford Diffraction, 2010 ▶) *T*_min_ = 0.821, *T*_max_ = 1.000
19533 measured reflections
3214 independent reflections
1988 reflections with *I* > 2σ(*I*)
*R*_int_ = 0.057

#### Refinement

*R*[*F* ^2^ > 2σ(*F* ^2^)] = 0.059
*wR*(*F* ^2^) = 0.154
*S* = 1.04
3214 reflections
253 parameters
6 restraints
H atoms treated by a mixture of independent and constrained refinement
Δρ_max_ = 0.24 e Å^−3^
Δρ_min_ = −0.40 e Å^−3^

### 

Data collection: *CrysAlis PRO* (Oxford Diffraction, 2010 ▶); cell refinement: *CrysAlis PRO*; data reduction: *CrysAlis PRO*; program(s) used to solve structure: *SHELXS97* (Sheldrick, 2008 ▶); program(s) used to refine structure: *SHELXL97* (Sheldrick, 2008 ▶); molecular graphics: *ORTEP-3* (Farrugia, 1997 ▶); software used to prepare material for publication: *PLATON* (Spek, 2009 ▶).

## Supplementary Material

Crystal structure: contains datablock(s) I, New_Global_Publ_Block. DOI: 10.1107/S1600536812042316/gk2523sup1.cif

Structure factors: contains datablock(s) I. DOI: 10.1107/S1600536812042316/gk2523Isup2.hkl

Supplementary material file. DOI: 10.1107/S1600536812042316/gk2523Isup3.cml

Additional supplementary materials:  crystallographic information; 3D view; checkCIF report

## Figures and Tables

**Table 1:** Hydrogen-bond geometry (Å, °)

*D*—H⋯*A*	*D*—H	H⋯*A*	*D*⋯*A*	*D*—H⋯*A*
O4—H41⋯O3^i^	0.85 (4)	1.78 (4)	2.626 (3)	174 (4)
C15—H15⋯O3^ii^	0.93	2.58	3.392 (3)	146
C17—H17*A*⋯F11*A* ^iii^	0.96	2.41	3.135 (14)	132

Symmetry codes: (i) 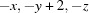; (ii) 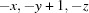; (iii) 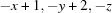.

**Fig. 1.**
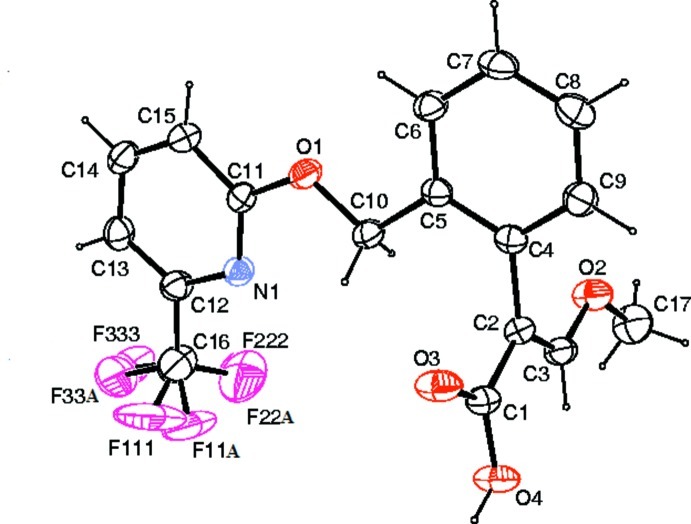
ORTEP view of the molecule with the atom-labeling scheme. The thermal ellipsoids are drawn at the 40% probability level. H atoms are shown as small spheres of arbitrary radii.

**Fig. 2.**
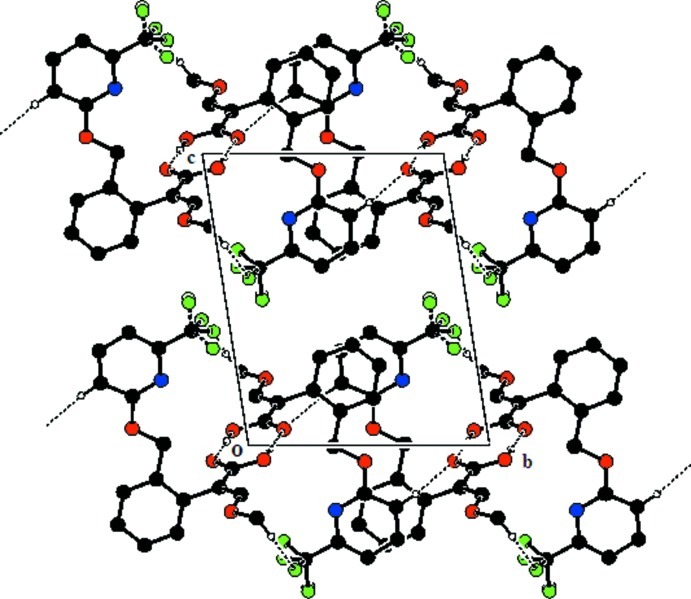
The packing arrangement of molecules viewed down the a axis. The dotted lines show intermolecular C—H···O, O—H···O and C—H···F hydrogen bonds.

## supplementary crystallographic information

### Comment

The above compound is the acid metabolite of picoxystrobin, which is a systemic fungicide of strobilurin group with broad spectrum bio-efficacy against various diseases of economically important agricultural crops (Balba, 2007; Ammermann *et al.*, 2000).

In (I) (Fig.1), all bond lengths and angles are normal and correspond to those observed in the related structure (Kant *et al.*, 2012). The dihedral angle between the two aromatic rings is 1.93 (9)°. The propenoic acid fragment is nearly perpendicular to the attached benzene ring [dihedral angle 82.6 (1)°]. The two nearly planar fragments, 2-(benzyloxy)-3-(trifluoromethyl)pyridine unit (r.m.s. deviation 0.016Å) and (*E*)-3-methoxyprop2-enoic unit (r.m.s. deviation 0.004Å) form dihedral angle of 84.19 (7)°. The F atoms of the trifluoromethyl group were refined as disordered over two sets of sites with occupancies of 0.53 (4)/0.47 (4). In the crystal, O—H···O hydrogen bonds link molecules to form dimers (Table 1). Dimers are further connected by C—H···O and C—H···F hydrogen bonds into (001) layers (Fig. 2). The crystal structure is further stabilized by π–π interactions between the pyridine ring (C11—C15/N1) of the molecule at (*x*, *y*, *z*) and the benzene ring of an inversion related molecule at(1 - *x*, 1 - *y*, - *z*) [centroid separation = 3.768 (2) Å, interplanar spacing = 3.437 Å and centroid shift = 1.54 Å].

### Experimental

Picoxystrobin (0.353 g, 0.001 mol) was dissolved in 5 ml of acetone and to it 5 ml of 1 N NaOH solution was added. The reaction mixture was refluxed at 343 K for 6 h, and then cooled. The compound was precipitated by neutralizing with 1 N HCl solution. The precipitated compound was dissolved in methanol and crystallized by the process of slow evaporation.(m.p. 415 K).

### Refinement

H atom bonded to O atom was located in a difference map and refined freely. Other H atoms were positioned geometrically and were treated as riding on their parent C atoms, with C—H distances of 0.93–0.97 Å and with *U*_iso_(H) = 1.2*U*_eq_(C) or 1.5*U*_eq_(methyl C). In the refinement process restraints were imposed on C-F distances of the disordered molecular fragments.

### Crystal data

**Table:**

C_17_H_14_F_3_NO_4_	*Z* = 2
*M**_r_* = 353.29	*F*(000) = 364
Triclinic, *P*1	*D*_x_ = 1.437 Mg m^−^^3^
Hall symbol: -P 1	Melting point: 415 K
*a* = 7.4701 (4) Å	Mo *K*α radiation, λ = 0.71073 Å
*b* = 10.1619 (5) Å	Cell parameters from 7723 reflections
*c* = 11.8219 (5) Å	θ = 3.5–29.0°
α = 94.721 (4)°	µ = 0.13 mm^−^^1^
β = 100.079 (4)°	*T* = 293 K
γ = 110.685 (5)°	Plate, colourless
*V* = 816.42 (7) Å^3^	0.3 × 0.2 × 0.2 mm

### Data collection

**Table:**

Oxford Diffraction Xcalibur Sapphire3 diffractometer	3214 independent reflections
Radiation source: fine-focus sealed tube	1988 reflections with *I* > 2σ(*I*)
Graphite monochromator	*R*_int_ = 0.057
Detector resolution: 16.1049 pixels mm^-1^	θ_max_ = 26.0°, θ_min_ = 3.5°
ω scan	*h* = −9→9
Absorption correction: multi-scan (*CrysAlis PRO*; Oxford Diffraction, 2010)	*k* = −12→12
*T*_min_ = 0.821, *T*_max_ = 1.000	*l* = −14→14
19533 measured reflections	

### Refinement

**Table:**

Refinement on *F*^2^	Primary atom site location: structure-invariant direct methods
Least-squares matrix: full	Secondary atom site location: difference Fourier map
*R*[*F*^2^ > 2σ(*F*^2^)] = 0.059	Hydrogen site location: inferred from neighbouring sites
*wR*(*F*^2^) = 0.154	H atoms treated by a mixture of independent and constrained refinement
*S* = 1.04	*w* = 1/[σ^2^(*F*_o_^2^) + (0.0666*P*)^2^ + 0.1912*P*] where *P* = (*F*_o_^2^ + 2*F*_c_^2^)/3
3214 reflections	(Δ/σ)_max_ < 0.001
253 parameters	Δρ_max_ = 0.24 e Å^−^^3^
6 restraints	Δρ_min_ = −0.40 e Å^−^^3^

### Special details

**Table:**

Experimental. *CrysAlis PRO*, Oxford Diffraction Ltd., Version 1.171.34.40 (release 27–08-2010 CrysAlis171. NET) (compiled Aug 27 2010,11:50:40) Empirical absorption correction using spherical harmonics, implemented in SCALE3 ABSPACK scaling algorithm.
Geometry. All e.s.d.'s (except the e.s.d. in the dihedral angle between two l.s. planes) are estimated using the full covariance matrix. The cell e.s.d.'s are taken into account individually in the estimation of e.s.d.'s in distances, angles and torsion angles; correlations between e.s.d.'s in cell parameters are only used when they are defined by crystal symmetry. An approximate (isotropic) treatment of cell e.s.d.'s is used for estimating e.s.d.'s involving l.s. planes.
Refinement. Refinement of *F*^2^ against ALL reflections. The weighted *R*-factor *wR* and goodness of fit *S* are based on *F*^2^, conventional *R*-factors *R* are based on *F*, with *F* set to zero for negative *F*^2^. The threshold expression of *F*^2^ > σ(*F*^2^) is used only for calculating *R*-factors(gt) *etc*. and is not relevant to the choice of reflections for refinement. *R*-factors based on *F*^2^ are statistically about twice as large as those based on *F*, and *R*- factors based on ALL data will be even larger.

### Fractional atomic coordinates and isotropic or equivalent isotropic displacement parameters (Å^2^)

**Table:**

	*x*	*y*	*z*	*U*_iso_*/*U*_eq_	Occ. (<1)
O1	0.3119 (3)	0.52862 (18)	0.05675 (15)	0.0505 (5)	
N1	0.5088 (3)	0.6809 (2)	0.22310 (18)	0.0441 (5)	
O2	0.5218 (3)	0.88494 (19)	−0.22824 (18)	0.0579 (6)	
O3	−0.0083 (3)	0.84278 (19)	−0.05459 (18)	0.0578 (6)	
O4	0.2235 (3)	1.05406 (19)	−0.05040 (19)	0.0580 (6)	
C1	0.1465 (4)	0.9160 (3)	−0.0804 (2)	0.0403 (6)	
C2	0.2512 (4)	0.8515 (2)	−0.1454 (2)	0.0369 (6)	
C3	0.4172 (4)	0.9358 (3)	−0.1689 (2)	0.0437 (6)	
H3	0.4622	1.0333	−0.1434	0.052*	
C4	0.1601 (3)	0.6946 (2)	−0.1883 (2)	0.0358 (6)	
C5	0.1822 (3)	0.5959 (2)	−0.1174 (2)	0.0371 (6)	
C6	0.0916 (4)	0.4512 (3)	−0.1611 (2)	0.0436 (6)	
H6	0.1052	0.3847	−0.1142	0.052*	
C7	−0.0180 (4)	0.4060 (3)	−0.2733 (2)	0.0502 (7)	
H7	−0.0781	0.3090	−0.3016	0.060*	
C8	−0.0393 (4)	0.5030 (3)	−0.3437 (2)	0.0529 (7)	
H8	−0.1136	0.4720	−0.4194	0.063*	
C9	0.0500 (4)	0.6462 (3)	−0.3013 (2)	0.0477 (7)	
H9	0.0363	0.7118	−0.3492	0.057*	
C10	0.3053 (4)	0.6487 (3)	0.0043 (2)	0.0434 (6)	
H10A	0.4369	0.7114	0.0023	0.052*	
H10B	0.2486	0.7015	0.0491	0.052*	
C11	0.4141 (4)	0.5507 (3)	0.1669 (2)	0.0402 (6)	
C12	0.6087 (4)	0.6917 (3)	0.3322 (2)	0.0492 (7)	
C13	0.6148 (4)	0.5784 (3)	0.3857 (2)	0.0521 (7)	
H13	0.6848	0.5918	0.4617	0.062*	
C14	0.5133 (4)	0.4432 (3)	0.3227 (2)	0.0519 (7)	
H14	0.5142	0.3634	0.3558	0.062*	
C15	0.4128 (4)	0.4282 (3)	0.2127 (2)	0.0484 (7)	
H15	0.3444	0.3384	0.1685	0.058*	
C16	0.7204 (6)	0.8399 (4)	0.3923 (3)	0.0835 (12)	
C17	0.6970 (5)	0.9862 (3)	−0.2501 (3)	0.0698 (9)	
H17A	0.6635	1.0336	−0.3127	0.105*	
H17B	0.7790	0.9378	−0.2709	0.105*	
H17C	0.7662	1.0551	−0.1813	0.105*	
F111	0.591 (3)	0.888 (3)	0.429 (2)	0.173 (7)	0.47 (4)
F222	0.854 (19)	0.914 (18)	0.334 (12)	0.145 (5)	0.47 (4)
F333	0.848 (3)	0.846 (2)	0.4878 (15)	0.115 (6)	0.47 (4)
F11A	0.6329 (17)	0.9292 (9)	0.3891 (12)	0.105 (4)	0.53 (4)
F22A	0.845 (17)	0.916 (16)	0.331 (11)	0.145 (5)	0.53 (4)
F33A	0.806 (3)	0.847 (2)	0.5018 (9)	0.109 (5)	0.53 (4)
H41	0.160 (6)	1.088 (4)	−0.012 (3)	0.101 (13)*	

### Atomic displacement parameters (Å^2^)

**Table:**

	*U*^11^	*U*^22^	*U*^33^	*U*^12^	*U*^13^	*U*^23^
O1	0.0606 (12)	0.0338 (10)	0.0482 (11)	0.0123 (9)	0.0007 (9)	0.0076 (8)
N1	0.0467 (13)	0.0386 (12)	0.0423 (13)	0.0132 (10)	0.0035 (10)	0.0059 (10)
O2	0.0551 (12)	0.0447 (11)	0.0786 (14)	0.0159 (10)	0.0339 (11)	0.0074 (10)
O3	0.0480 (12)	0.0373 (10)	0.0867 (15)	0.0104 (9)	0.0282 (11)	−0.0015 (10)
O4	0.0540 (13)	0.0321 (10)	0.0882 (16)	0.0125 (9)	0.0301 (11)	−0.0030 (10)
C1	0.0405 (15)	0.0310 (13)	0.0460 (15)	0.0121 (12)	0.0054 (12)	0.0020 (11)
C2	0.0397 (14)	0.0312 (13)	0.0407 (14)	0.0141 (12)	0.0089 (11)	0.0069 (11)
C3	0.0494 (16)	0.0353 (14)	0.0505 (16)	0.0192 (13)	0.0141 (13)	0.0072 (12)
C4	0.0308 (13)	0.0357 (13)	0.0418 (14)	0.0132 (11)	0.0108 (11)	0.0020 (11)
C5	0.0354 (14)	0.0349 (14)	0.0404 (14)	0.0113 (11)	0.0124 (11)	0.0030 (11)
C6	0.0437 (15)	0.0334 (14)	0.0502 (16)	0.0090 (12)	0.0133 (13)	0.0063 (12)
C7	0.0482 (17)	0.0356 (15)	0.0546 (18)	0.0044 (13)	0.0108 (13)	−0.0078 (13)
C8	0.0487 (17)	0.0549 (18)	0.0455 (16)	0.0164 (14)	−0.0004 (13)	−0.0070 (14)
C9	0.0508 (17)	0.0488 (16)	0.0450 (16)	0.0235 (14)	0.0052 (13)	0.0059 (13)
C10	0.0475 (16)	0.0315 (13)	0.0454 (15)	0.0088 (12)	0.0079 (12)	0.0068 (11)
C11	0.0397 (15)	0.0384 (14)	0.0426 (15)	0.0132 (12)	0.0111 (12)	0.0098 (12)
C12	0.0532 (17)	0.0442 (16)	0.0490 (17)	0.0199 (14)	0.0048 (13)	0.0062 (13)
C13	0.0547 (18)	0.0600 (19)	0.0454 (16)	0.0266 (15)	0.0069 (13)	0.0147 (14)
C14	0.0585 (18)	0.0467 (17)	0.0587 (19)	0.0244 (15)	0.0173 (15)	0.0213 (14)
C15	0.0537 (17)	0.0372 (15)	0.0540 (18)	0.0161 (13)	0.0123 (14)	0.0086 (12)
C16	0.104 (3)	0.058 (2)	0.061 (2)	0.014 (2)	−0.017 (2)	0.0047 (18)
C17	0.059 (2)	0.068 (2)	0.086 (2)	0.0171 (17)	0.0335 (18)	0.0197 (18)
F111	0.267 (14)	0.108 (10)	0.142 (12)	0.136 (10)	−0.061 (8)	−0.060 (8)
F222	0.162 (8)	0.077 (4)	0.114 (6)	−0.033 (5)	−0.012 (6)	0.011 (3)
F333	0.121 (8)	0.060 (6)	0.101 (9)	0.002 (5)	−0.067 (8)	0.006 (7)
F11A	0.144 (7)	0.039 (3)	0.111 (6)	0.042 (5)	−0.035 (4)	−0.007 (3)
F22A	0.162 (8)	0.077 (4)	0.114 (6)	−0.033 (5)	−0.012 (6)	0.011 (3)
F33A	0.166 (11)	0.090 (7)	0.046 (4)	0.043 (7)	−0.021 (5)	−0.009 (4)

### Geometric parameters (Å, º)

**Table:**

O1—C11	1.346 (3)	C8—H8	0.9300
O1—C10	1.425 (3)	C9—H9	0.9300
N1—C11	1.313 (3)	C10—H10A	0.9700
N1—C12	1.347 (3)	C10—H10B	0.9700
O2—C3	1.336 (3)	C11—C15	1.396 (3)
O2—C17	1.432 (3)	C12—C13	1.370 (4)
O3—C1	1.240 (3)	C12—C16	1.482 (4)
O4—C1	1.304 (3)	C13—C14	1.384 (4)
O4—H41	0.85 (4)	C13—H13	0.9300
C1—C2	1.457 (3)	C14—C15	1.350 (4)
C2—C3	1.326 (3)	C14—H14	0.9300
C2—C4	1.497 (3)	C15—H15	0.9300
C3—H3	0.9300	C16—F11A	1.291 (7)
C4—C9	1.387 (3)	C16—F33A	1.324 (8)
C4—C5	1.394 (3)	C16—F333	1.326 (9)
C5—C6	1.392 (3)	C16—F111	1.346 (10)
C5—C10	1.505 (3)	C16—F222	1.350 (10)
C6—C7	1.376 (4)	C16—F22A	1.350 (9)
C6—H6	0.9300	C17—H17A	0.9600
C7—C8	1.375 (4)	C17—H17B	0.9600
C7—H7	0.9300	C17—H17C	0.9600
C8—C9	1.375 (4)		
			
C11—O1—C10	118.71 (19)	O1—C10—H10A	110.0
C11—N1—C12	115.5 (2)	C5—C10—H10A	110.0
C3—O2—C17	117.1 (2)	O1—C10—H10B	110.0
C1—O4—H41	115 (3)	C5—C10—H10B	110.0
O3—C1—O4	121.7 (2)	H10A—C10—H10B	108.4
O3—C1—C2	121.4 (2)	N1—C11—O1	120.1 (2)
O4—C1—C2	116.9 (2)	N1—C11—C15	124.4 (2)
C3—C2—C1	118.3 (2)	O1—C11—C15	115.5 (2)
C3—C2—C4	123.2 (2)	N1—C12—C13	124.6 (3)
C1—C2—C4	118.4 (2)	N1—C12—C16	114.5 (2)
C2—C3—O2	122.0 (2)	C13—C12—C16	120.9 (3)
C2—C3—H3	119.0	C12—C13—C14	117.7 (3)
O2—C3—H3	119.0	C12—C13—H13	121.1
C9—C4—C5	119.2 (2)	C14—C13—H13	121.1
C9—C4—C2	119.2 (2)	C15—C14—C13	119.3 (3)
C5—C4—C2	121.6 (2)	C15—C14—H14	120.3
C6—C5—C4	119.3 (2)	C13—C14—H14	120.3
C6—C5—C10	121.6 (2)	C14—C15—C11	118.4 (3)
C4—C5—C10	119.1 (2)	C14—C15—H15	120.8
C7—C6—C5	120.4 (2)	C11—C15—H15	120.8
C7—C6—H6	119.8	F11A—C16—C12	118.5 (5)
C5—C6—H6	119.8	F33A—C16—C12	113.2 (9)
C8—C7—C6	120.5 (2)	F333—C16—C12	112.1 (9)
C8—C7—H7	119.8	F111—C16—C12	106.9 (10)
C6—C7—H7	119.8	F222—C16—C12	112 (8)
C7—C8—C9	119.5 (3)	F22A—C16—C12	112 (7)
C7—C8—H8	120.2	O2—C17—H17A	109.5
C9—C8—H8	120.2	O2—C17—H17B	109.5
C8—C9—C4	121.1 (3)	H17A—C17—H17B	109.5
C8—C9—H9	119.4	O2—C17—H17C	109.5
C4—C9—H9	119.4	H17A—C17—H17C	109.5
O1—C10—C5	108.36 (19)	H17B—C17—H17C	109.5
			
O3—C1—C2—C3	−179.2 (2)	C12—N1—C11—O1	−179.1 (2)
O4—C1—C2—C3	0.5 (4)	C12—N1—C11—C15	−0.2 (4)
O3—C1—C2—C4	4.4 (4)	C10—O1—C11—N1	−1.5 (3)
O4—C1—C2—C4	−175.9 (2)	C10—O1—C11—C15	179.5 (2)
C1—C2—C3—O2	−179.9 (2)	C11—N1—C12—C13	−0.6 (4)
C4—C2—C3—O2	−3.7 (4)	C11—N1—C12—C16	177.8 (3)
C17—O2—C3—C2	179.1 (2)	N1—C12—C13—C14	0.9 (4)
C3—C2—C4—C9	−80.6 (3)	C16—C12—C13—C14	−177.4 (3)
C1—C2—C4—C9	95.6 (3)	C12—C13—C14—C15	−0.3 (4)
C3—C2—C4—C5	99.8 (3)	C13—C14—C15—C11	−0.5 (4)
C1—C2—C4—C5	−84.0 (3)	N1—C11—C15—C14	0.8 (4)
C9—C4—C5—C6	−0.7 (3)	O1—C11—C15—C14	179.7 (2)
C2—C4—C5—C6	178.9 (2)	N1—C12—C16—F11A	47.7 (9)
C9—C4—C5—C10	178.7 (2)	C13—C12—C16—F11A	−133.9 (8)
C2—C4—C5—C10	−1.7 (3)	N1—C12—C16—F33A	175.8 (12)
C4—C5—C6—C7	0.3 (4)	C13—C12—C16—F33A	−5.8 (13)
C10—C5—C6—C7	−179.1 (2)	N1—C12—C16—F333	−166.4 (15)
C5—C6—C7—C8	0.1 (4)	C13—C12—C16—F333	12.1 (16)
C6—C7—C8—C9	0.1 (4)	N1—C12—C16—F111	78.5 (15)
C7—C8—C9—C4	−0.5 (4)	C13—C12—C16—F111	−103.0 (16)
C5—C4—C9—C8	0.8 (4)	N1—C12—C16—F222	−60 (8)
C2—C4—C9—C8	−178.8 (2)	C13—C12—C16—F222	119 (9)
C11—O1—C10—C5	−179.7 (2)	N1—C12—C16—F22A	−56 (8)
C6—C5—C10—O1	1.9 (3)	C13—C12—C16—F22A	123 (8)
C4—C5—C10—O1	−177.5 (2)		

### Hydrogen-bond geometry (Å, º)

**Table:**

*D*—H···*A*	*D*—H	H···*A*	*D*···*A*	*D*—H···*A*
O4—H41···O3^i^	0.85 (4)	1.78 (4)	2.626 (3)	174 (4)
C15—H15···O3^ii^	0.93	2.58	3.392 (3)	146
C17—H17*A*···F11*A*^iii^	0.96	2.41	3.135 (14)	132

Symmetry codes: (i) −*x*, −*y*+2, −*z*; (ii) −*x*, −*y*+1, −*z*; (iii) −*x*+1, −*y*+2, −*z*.
